# A prospective study on sex differences in functional capacity, quality of life and prognosis in patients with heart failure

**DOI:** 10.1097/MD.0000000000029795

**Published:** 2022-06-30

**Authors:** Yiming Ma, Yunke Shi, Wenfang Ma, Dan Yang, Zhao Hu, Mingqiang Wang, Xingyu Cao, Chaoyue Zhang, Xiang Luo, Shulin He, Min Zhang, Yong Duan, Hongyan Cai

**Affiliations:** a Cardiology Department, the First Affiliated Hospital of Kunming Medical University, Kunming, Yunnan, China; b Cardiology Department, People’s Hospital of Chuxiong Yi Autonomous Prefecture, Chuxiong, Yunnan, China; c Yunnan Key Laboratory of Laboratory Medicine, Yunnan Institute of Experimental Diagnosis, Department of Clinical Laboratory, the First Affiliated Hospital of Kunming Medical University, Kunming, China.

**Keywords:** functional capacity, heart failure, prognosis, quality of life, sex

## Abstract

Due to the lack of evidence and inconsistency of sex differences in Heart failure (HF) in the Chinese population, this study aimed to compare sex differences in functional capacity and quality of life (QoL) between women and men after standard HF medications therapies, and analyze whether sex differences were associated with the composite endpoints of all-cause mortality or HF-related hospitalization and cardiac event-free survival rate in Chinese patients with HF.

**Methods:**

This was a 1-year longitudinal study. Participants included patients with HF from March 2017 to December 2018. At baseline and followed up at 1, 6, and 12 months later, functional capacity was assessed by 6-minute walk testing (6MWT), QoL was measured with the Kansas City Cardiomyopathy Questionnaire (KCCQ) and EuroQoL five dimensions (EQ-5D). The Cox proportional hazards model and Kaplan-Meier curves were used to determine sex differences in subsequent outcomes. The Cox proportional hazards model was used to identify the risk factors for composite endpoints. Kaplan-Meier curves were used to compare survival.

**Results:**

All patients were assigned to either men group (n = 94) or women group (n = 60). Longitudinal follow-ups showed a continuously increasing in 6MWT, Kansas City Cardiomyopathy Questionnaire overall score, EQ-5D visual analogue scale, and EQ-5D Index score in both groups (all *P* < 0.001); however, women reported a lower level of all parameters at the 1, 6, and 12 months follow-ups (all *P* < 0.05). In addition, women had a higher risk of all-cause mortality or HF-related hospitalization and a lower cardiac event-free survival rate than men (log-rank test, *P* = 0.027).

**Conclusion:**

Women reported worse functional capacity, QoL, and prognosis than men in a sample of Chinese patients with HF. Our findings highlight the importance of paying attention to sex differences in HF.

## 1. Introduction

Heart failure (HF) affects over 4.2 million people in China, with an estimated prevalence of 1.3% and remains serious public healthcare burden because of high hospitalization burden and mortality.^[1–3]^ Patients with HF have severe physical symptoms, high medical expenses, and terrible psychological activities such as anxiety and depression, which has seriously affected their quality of life (QoL) and exercise capacity.^[4]^

Although recent studies have demonstrated women with HF experienced poorer QoL as compared to men.^[5,6]^ Very little literature is available on the effect of sex differences in functional capacity, quality of life, and prognosis of patients with HF in Chinese population. Lower QoL in HF is associated with increased hospitalizations and mortality.^[7,8]^ Thus, it appears to be particularly true for women, who experience higher rates of death and HF hospitalizations than men.^[5,9]^ However, Previous studies have reported either men had higher hospitalization rates for HF and in-hospital mortality across virtually all ages,^[10]^ or similar in-hospital mortality rates between the sexes.^[11,12]^ The inconsistency of these findings makes it difficult for us to understand sex differences of HF and implies the need for more clinical trials from different regions and races to verify this point of view. An in-depth understanding of these sex differences may help choose treatment strategies and achieve precise treatment in clinical work. Only a recent retrospective study has reported sex-related differences in the quality of life and short-term mortality rates in Chinese patients with HF, and the clinical trials evidence on sex differences in HF is insufficient due to the limitations of selection bias and information bias in retrospective studies.^[13]^

Therefore, we conducted a single-center prospective cohort study to compare differences in the functional capacity and QoL between women and men after standard HF medications therapies at different follow-up points. Moreover, we analyzed whether sex differences were associated with the composite endpoints of all-cause mortality or HF-related hospitalization and cardiac event-free survival rate in patients with HF.

## 2. Methods

### 2.1. Study population

From March 2017 to December 2018, a total of 164 hospitalized patients who were diagnosed with HF at the First Affiliated Hospital of Kunming Medical University were enrolled in this study, regardless of EF and etiology. The study protocol was approved by the Institutional Review Board of the First Affiliated Hospital of Kunming Medical University and conforms to the Declaration of Helsinki and its subsequent revisions. All patients involved provided informed consent before enrolling in the study. HF was diagnosed based on standard guideline criteria.^[14]^ The main inclusion criteria were aged ≥18 years with signs and/or symptoms of new-onset or worsening heart failure and had objective evidence of cardiac dysfunction.

The exclusion criteria were: heart transplant recipient or admitted for cardiac transplantation; implantable cardioverter defibrillator and/or cardiac resynchronization therapy; acute myocarditis or hypertrophic obstructive; restrictive, or constrictive cardiomyopathy; acute coronary syndrome or stroke; septicemia; usage of tricyclic antidepressants, anxiolytics, or other central nervous system medications.

### 2.2. Study plan

After having obtained written informed consent, the following assessments were performed: demographics (including medication), a medical history and physical examination, and blood draw of plasma and serum, a 12-lead resting ECG and echocardiogram. All patients were treated with maximum targeted doses with beta-blockers, ACEI (angiotensin-converting enzyme inhibitor)/ARB (angiotensin receptor blocker), and/or diuretics according to the European Society of Cardiology guidelines except for patients with related drug contraindications.^[14,15]^

We used 6MWT, Kansas City Cardiomyopathy Questionnaire (KCCQ) and EQ-5D to compare functional capacity and quality of life at 1, 6, and 12 months with baseline in men and women: (1) the 6MWT is a simple, safe, inexpensive, widely available and well-tolerated test for assessing the functional capacity of patients with HF in daily clinical practice.^[16–18]^ It also has been used as a predictor of mortality in patients with HF.^[19]^ The 6MWT should be conducted on a flat, straight indoor corridor usually at least 300 cm long.^[20]^ The patients were told to be calm, to walk continuously if possible, and to cover as much ground as possible in 6 minutes, but they were allowed to stop or slow down if necessary. The total distance achieved was recorded at the end of the test. (2) The 12-item abbreviated version of the KCCQ is specifically designed and validated for health-related QoL assessment in patients with HF, including 4 domains (physical limitation, symptom frequency, quality of life, and social limitation).^[21,22]^ The EQ-5D questionnaire is a widely used for measuring generic health status consisting of 2 parts: the Index score and the Visual Analogue Scale (VAS). The Index score covers 5 domains of health (mobility, self-care, activity, pain/discomfort, and anxiety/depression), where the patients are asked to rate the severity of each domain in 5 levels. The VAS is a vertical type of measure from 0 (worst QoL) to 100 (best QoL).^[23]^ The higher KCCQ and EQ-5D score indicate the better the health status of subjects.

**Figure 1.**
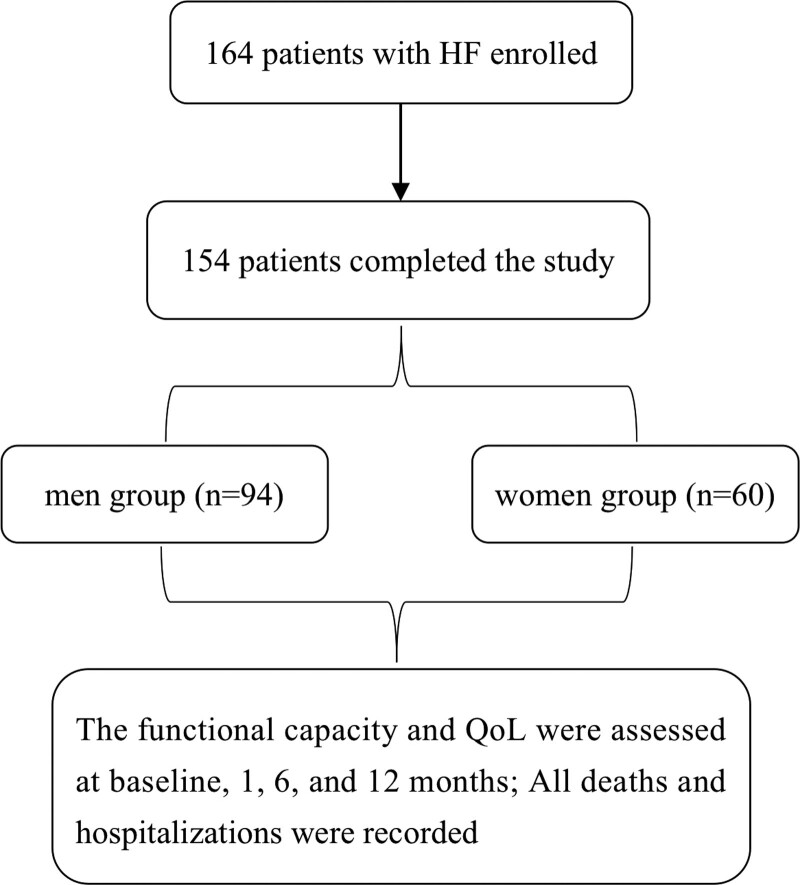
Study flow.

In addition, the composite endpoints including all-cause mortality and HF-related hospitalization were recorded during 1-year follow-up. All clinical outcomes were collected by standard clinic follow-up or telephone contact until the end of the study (Fig. 1).

### 2.3. Statistical analysis

Normally distributed continuous variables are presented as means ± standard deviation and nonnormally distributed variables as median (interquartile range, IQR), and categorical data are presented as frequencies (percentages). The baseline group comparisons were made using *t*-tests, Chi-square tests, and Mann-Whitney U tests as appropriate. Differences in 6MWT, KCCQ, and EQ-5D score were compared with the baseline by using repeated measures 1-way analysis of variance (ANOVA), followed by post hoc Dunnett’s multiple comparison test. Multivariable analyses with the Cox proportional-hazards model were used to estimate the simultaneous effects of prognostic factors on composite endpoints. The cumulative survival was plotted and compared using Kaplan–Meier curves and log-rank test. Significance level was set at *P*<0.05. Statistical analyses were performed using IBM SPSS Statistics for Windows, version 26.

## 3. Results

### 3.1. Baseline characteristics

Of the 164 patients included in the study, 10 patients were lost to follow-up. Complete data were obtained for 154 patients, 94 of whom were men and the remaining 60 patients were women. Baseline characteristics of men and women are displayed in Table 1. At baseline, age, BMI, heart rate, systolic blood pressure, and diastolic blood pressure were similar between men and women. Women were shorter (156.23 ± 5.73 vs 167.27 ± 6.85, *P* < 0.001), had slightly higher left ventricular EF (48.25 ± 19.53% vs 41.79 ± 16.96%, *P* = 0.037), and had less atrial fibrillation or flutter aetiology (*P* = 0.036). Notably, there was no significant sex difference in New York Heart Association (NYHA) class (*P* = 0.563), and B-type natriuretic peptide (BNP) (*P* = 0.058). Also, there was no significant sex difference in the fraction of target dose of ACEIs/ARBs, beta-blockers or diuretics.

**Table 1:** Baseline characteristics.

Characteristic	Men	Women	P
No. of subjects	94	60	
Demographics			
Age, years	60.43 ± 13.25	61.05 ± 13.40	0.777
BMI, kg/m^2^	24.40 ± 4.52	23.15 ± 3.99	0.080
Height, cm	167.27 ± 6.85	156.23 ± 5.73	<0.001
Clinical assessment			
Heart rate, bpm	91.34 ± 18.66	91.00 ± 23.56	0.921
Systolic blood pressure, mm Hg	122.07 ± 22.84	119.35 ± 20.87	0.457
Diastolic blood pressure, mm Hg	80.54 ± 15.18	75.83 ± 19.22	0.093
NYHA class, n (%)			0.563
II	17 (18.1)	10 (16.7)	
III	57 (60.6)	41 (68.3)	
IV	20 (21.3)	9 (15)	
LVEF, %	41.79 ± 16.96	48.25 ± 19.53	0.037
Medical history			
Hypertension: n (%)	45 (47.9)	32 (53.3)	0.509
Atrial fibrillation or flutter: n (%)	28 (29.8)	9 (15)	0.036
Ischemic heart disease: n (%)	32 (34)	17 (28.3)	0.458
Diabetes mellitus: n (%)	21 (22.3)	6 (10.0)	0.050
Chronic obstructive pulmonary disease: n (%)	10 (10.6)	1 (1.7)	0.074
Laboratory biomarkers			
BNP, pg/mL	1087.78 [513.25–1961.52]	786.00 [286.50–1498.55]	0.058
Medication, n (%)			
ACEI/ARBs	79 (84.0)	51 (85.0)	0.873
Beta-blocker	86 (91.5)	53 (88.3)	0.519
Diuretics	86 (91.5)	54 (90.0)	0.754

Continuous variables are presented as means ± standard deviation when normally distributed, or median [interquartile range] for nonnormally distributed variables. Categorical variables are shown as n (%).

ARB = angiotensin receptor blocker, ACEI = angiotensin-converting enzyme inhibitor, BMI = body mass index, BNP = B-type natriuretic peptide, LVEF = left ventricular ejection fraction, NYHA = New York Heart Association.

### 3.2. Changes in functional capacity and QoL outcomes

Functional capacity, evaluated using 6MWT, in men and women, respectively, improved significantly from 1 to 12 months compared with baseline (*P* < 0.001). A comparison of 6MWT between the men and women groups showed no significant difference walking distance at baseline. (269.21 ± 77.82 vs 248.75 ± 67.32, *P* = 0.122). However, women had a shorter walking distance than men at 1, 6, and 12 months (all *P* < 0.05) (Table 2).

**Table 2:** Comparison of functional capacity and QoL outcomes.

Groups	Baseline	1 month	6 months	12 months
6MWT				
Men	269.21 ± 77.82	320.87 ± 83.77*	364.21 ± 86.23*	430.15 ± 75.76*
Women	248.75 ± 67.32	285.96 ± 63.87*	326.18 ± 58.53*	394.12 ± 40.93*
*P*-value	0.122	0.012	0.006	0.002
KCCQ				
Men	43.75 ± 18.63	52.25 ± 22.13*	64.01 ± 14.78*	68.81 ± 13.67*
Women	40.96 ± 20.11	43.86 ± 21.32*	58.11 ± 14.16*	63.63 ± 12.87*
*P*-value	0.415	0.032	0.024	0.031
EQ-5D VAS				
Men	61.43 ± 10.11	72.70 ± 10.82*	75.74 ± 10.70*	80.37 ± 9.50*
Women	58.33 ± 14.96	67.84 ± 12.42*	70.60 ± 11.68*	75.20 ± 14.84*
*P*-value	0.154	0.018	0.010	0.015
EQ-5D Index				
Men	0.62 ± 0.17	0.73 ± 0.15*	0.80 ± 0.10*	0.87 ± 0.11*
Women	0.57 ± 0.17	0.67 ± 0.15*	0.74 ± 0.12*	0.79 ± 0.15*
*P*-value	0.109	0.027	0.002	0.001

* *P* < 0.001, compared with baseline; *P*-value, men vs women.

6MWT = 6-minute walk test, EQ-5D = EuroQoL 5 dimensions, KCCQ = Kansas City Cardiomyopathy Questionnaire, VAS = visual analogue scale.

Distributions of overall QoL measures at baseline and their change after 1, 6, 12 months followed a similar trend and are showed in Table 2. Compared all scores measured at baseline, QoL evaluated by KCCQ overall score, EQ-5D VAS and EQ-5D Index score improved significantly at the 1-, 6-, and 12-month follow ups in both men and women group (all *P* < .001). Although women reported similar QoL score with men at baseline as assessed with KCCQ overall score (43.75 ± 18.63 vs 40.96 ± 20.11, *P* = 0.415), EQ-5D VAS score (61.43 ± 10.11 vs 58.33 ± 14.96, *P* = 0.154), and EQ-5D Index score (0.62 ± 0.17 vs 0.57 ± 0.17, *P* = 0.109), women had a lower KCCQ and EQ-5D score than men during subsequent 1-year follow-up period (all *P* < 0.05)

### 3.3. Sex differences in composite endpoints and cardiac event-free survival rate

**Figure 2.**
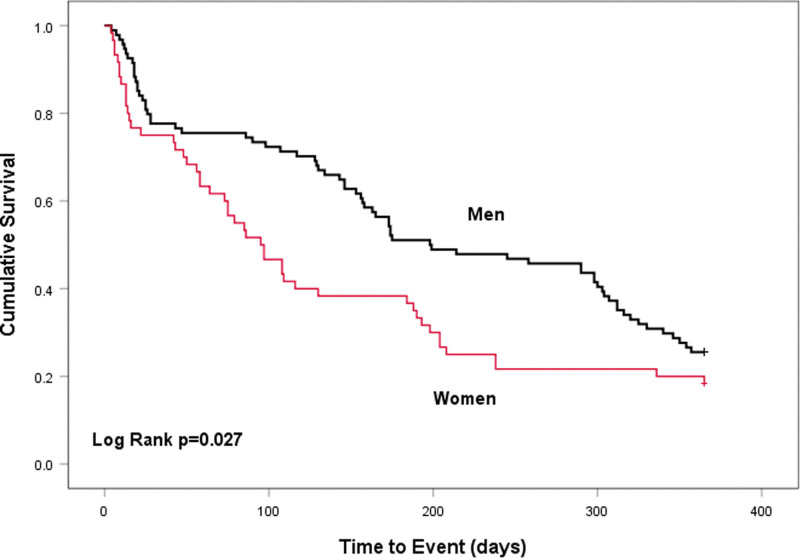
Kaplan Meier curves were used to study cardiac event-free survival rate at different sex.

At the end of the follow-up period, 19 patients died and 100 patients were first hospitalized for HF-related reasons. In the univariate Cox regression model, the sex differences were associated with the composite endpoints of all-cause mortality or first HF-related hospitalization (HR: 1.509, 95% CI:1.045–2.178, *P* = 0.028) (Table 3). In the multivariate model, the sex differences was also associated with the composite endpoints (Table 3). Women had a 1.7-fold high risk of all-cause mortality or HF-related hospitalization (HR: 1.718, 95% CI:1.168–2.555, *P* = 0.006), after adjusting for age, BMI, NYHA class, blood pressure, and LVEF. Among the covariates age was a risk factor (HR: 1.028, 95% CI: 1.012–1.044, *P* = 0.001). In Figure 2, Kaplan Meier survival curves showed that the women had a lower cardiac event-free survival rate within 1 year of follow-up (log-rank test, *P*=0.027).

**Table 3:** Univariate and Multivariable Cox regression analysis

	Hazard ratio	*P*-value	95% Confidence interval
Univariate model			
women (vs men)	1.509	0.028	1.045, 2.178
Multivariable model			
women (vs men)	1.728	0.006	1.168, 2.555
age	1.028	0.001	1.012, 1.044
NYHA Class III (vs II)	1.024	0.932	0.598, 1.752
NYHA Class IV (vs II)	1.666	0.113	0.886, 3.134
BMI, kg/m^2^	1.021	0.364	0.976, 1.068
Systolic blood pressure	0.992	0.23	0.980, 1.005
Diastolic blood pressure	1.004	0.579	0.989, 1.020
LVEF, %	0.992	0.221	0.980, 1.005

BMI = body mass index, LVEF = left ventricular ejection fraction, NYHA = New York Heart Association.

## 4. Discussion

Due to the lack of evidence of sex differences in the Chinese population with HF, this study aimed to compare the differences in functional capacity and QoL between women and men, and analyze whether sex differences were associated with the composite endpoints in patients with HF. We found women reported worse functional capacity and QoL than men from 1 to 12 months compared with baseline in Chinese HF patients, although all these parameters have a significant improvement during subsequent 1-year follow-up period. In addition, women had higher risk of composite endpoints of all-cause mortality or HF-related hospitalization and lower cardiac event-free survival rate than men.

At 1-year follow-up, both men and women showed a similar improvement in functional capacity and QoL. Several potential contributions to the phenomenon include: Firstly, the improvement presented maximum opportunity at follow-up owing to patients were enrolled either with new-onset or worsening HF symptoms. Secondly, the majority of patients accepted standard HF drugs included ACEI/ARB and beta-blockers according to recent ESC guidelines.^[14]^ ACEI/ARB and beta-blockers are widely known to improve QoL,^[24–28]^ ejection fraction (EF),^[29,30]^ and functional capacity.^[24,27]^ Thirdly, patients enrolled in this study might have benefited from the close follow-up and clinical management even if there had no improvement in medication adherence. Hence, the findings from this study highlight the importance of regular follow-up and optimal management of patients after discharge, as QoL and functional capacity benefit is equally present in both men and women when managed according to guidelines.

Delaying the progress of HF, preventing cardiovascular events, and prolonging survival are recommended in current guidelines.^[14]^ Although compelling evidence and guidelines emphasized that the benefit of using maximum targeted doses with HF medications such as beta-blockers and ACEI/ARB strengthened over time, target doses are usually not achieved in clinical practice.^[31]^ A study published in the journal of JACC showed HF medications doses is lower than what is recommended by trials, and a proportion of patients receiving maximum target doses were low at 10.8% for ACEI/ARB, 18.7% for beta-blockers.^[32]^ A lower use of guideline recommended drugs in female with HF has been reported in series of studies which could lead a negative influence on the HF treatment.^[33,34]^ In our study, all patients involved were treated with maximum targeted doses with beta-blockers and ACEI/ARB except for patients with related drug contraindications. Also, baseline characteristics showed there was similar fraction of targeted dose of these HF drugs; however, our findings reported that women had a lower improvement of 6MWT, KCCQ overall score, EQ-5D VAS and EQ-5D Index score, and indicated women had a worse functional capacity and QoL although after standard HF therapy. Recently, health-related QoL has been shown to have prognostic relevance in patients with HF.^[7,8]^ Therefore, it is also important for us to recognize the sex differences in HF for prognostic assessment and personalized therapeutic strategies. When it comes to worse prognosis in women, our study showed women was associated with a higher risk composite endpoints of all-cause mortality or HF-related hospitalization than men, after controlling for demographic and clinical confoundings. Kaplan Meier survival curves indicated that women had lower cardiac event-free survival rate. The reason may be owing to the fact that women with HF are more likely to be older, have more comorbidities,^[35]^ and underrepresented in clinical trials,^[36]^ which makes physician worried about less effective and adverse drug reactions in female patients. It is noteworthy to mention that women are more likely to have HF with preserved EF, for which therapies are aimed only at alleviating symptoms.^[37]^ The difference of demographics and clinical characteristics may contribute to the outcome.

Interestingly, these sex differences in functional capacity, QoL and prognosis were observed during subsequent 1-year follow-up period in this study even though slightly higher left ventricular ejection in women and similar age, NYHA class, BNP, comorbidities, medication and 6MWT and QoL score at baseline between men and women. Additional attention should be paid to the role of hormones in modulating heart function and anatomy.^[38,39]^ To our best knowledge, menopause is related to fat mass, hypertension, endothelial function and cardiomyocyte fibrosis.^[40,41]^ Moreover, coronary microvascular dysfunction and endothelial inflammation plays a key role in female HF syndromes.^[41]^ Compared with men, women have smaller vessels^[42]^ and appear to be predisposed to poorer diastolic reserve and arterial compliance.^[43]^ This may further contribute to the impact of hypertension on diastolic function and exercise capacity. In addition, women with HF not only have greater physical limitations, but also much higher rates of anxiety and depression than men.^[44]^ Taken together, these findings suggest that women living with HF experience impairment in functional status, poorer QoL, and worse prognosis. Hence, our findings highlight the importance of sex-specific strategies to improve the prognosis of patients with HF. More HF clinical trials performed sex disaggregated analyses should be encouraged to show the sex-specific outcomes and efficacy.^[41]^ A national registry study of 167 US outpatient cardiology practices has showed women received less HF education and less device therapy in the outpatient cardiology setting than their male counterparts with HF; so more education is needed to improve rates of provision of guideline-recommended therapies in women.^[45]^ The patient-centered transitional care model improved discharge preparedness and quality of care transition in both men and women, particularly effective in women, implying more care for women could improve women’s poor HF prognosis.^[46]^ Therapeutic strategies should consider patient characteristics, including sex, rather than same approach. For example, women may benefit from an emphasis on caloric restriction, exercise training, and/or SGLT2 inhibitors.^[47]^ Further research is needed to identify sex differences in medical therapy and patient care to improve outcomes in women.

Our study has several potential limitations. First, our sample size was small, which may have also resulted in the inability to detect significant interactions between sex and treatment over serial time points. Future studies with a larger population and longer follow-up are required to validate the long-term consequences of sex difference in HF. Second, lack of effective therapies in preserved EF HF may confound outcomes, the influence of heart failure phenotype must be taken into account. Third, we are unable to comment on important changes in the characteristics or outcomes of patients during the study period owing to the short period of observation. Fourth, the single-center nature of our study potentially limits the heterogeneity of sample considered, which may cannot be extrapolated to other population elsewhere.

## 5. Conclusions

Women reported worse functional capacity and QoL than men compared with baseline in Chinese HF patients, although all these parameters have a significant improvement in both men and women during longitudinal 1-year follow-up period. In addition, women had higher risk of composite endpoints of all-cause mortality or HF-related hospitalization and lower cardiac event-free survival rate than men. These sex differences in functional capacity, QoL, and prognosis were observed in this study even though similar demographics and clinical characteristics. Our findings highlight the importance of paying attention to sex differences in HF, which may result in the improvement of outcomes in HF.

## Author contributions

Conceptualization: Hongyan Cai, Yong Duan

Data curation: Hongyan Cai, Xingyu Cao

Funding acquisition: Hongyan Cai, Zhao Hu

Investigation: Yiming Ma, Dan Yang, Mingqiang Wang, Shulin He, Xiang Luo

Methodology: Yiming Ma, Yunke Shi, Min Zhang, Chaoyue Zhang

Formal analysis: Wenfang Ma, Shulin He, Min Zhang

Supervision: Hongyan Cai, Yong Duan

Writing-original draft: Yiming Ma, Yunke Shi

Writing-review and editing: Zhao Hu, Min Zhang
